# Supplementary material for: A Critical View of the Application of the APEX Software (Aqueous Photochemistry of Environmentally-Occurring Xenobiotics) to Predict Photoreaction Kinetics in Surface Freshwaters
Source: Molecules. 2019 Dec 18;25(1):9. doi: 10.3390/molecules25010009 (PMC7017383; doi:10.3390/molecules25010009)
Supplement: Supplementary file 1 [file molecules-25-00009-s001.zip › 3.2.4_gcc-4.4.0.zip!/3.2.4_gcc-4.4.0/3.2.4_gcc-4.4.0/doc/octave/html/interpreter/Line-Properties.html]

Line Properties - Untitled


Next: Text Properties,
Previous: Axes Properties,
Up: Graphics Object Properties


---

##### 15.2.2.4 Line Properties

`xdata` `ydata` `zdata` `ldata` `udata` `xldata` `xudata`: The data to be plotted. The `ldata` and `udata` elements are for errorbars in the y direction, and the `xldata` and `xudata` elements are for errorbars in the x direction. `color`: The RGB color of the line, or a color name. See Colors. `linestyle` `linewidth`: See Line Styles. `marker` `markeredgecolor` `markerfacecolor` `markersize`: See Marker Styles. `keylabel`: The text of the legend entry corresponding to this line. Note that this property is not compatible with matlab and may be removed in a future version of Octave.
